# Targeted drug delivery of engineered mesenchymal stem/stromal-cell-derived exosomes in cardiovascular disease: recent trends and future perspectives

**DOI:** 10.3389/fbioe.2024.1363742

**Published:** 2024-03-15

**Authors:** Jian-Liang Pang, Hong Shao, Xiao-Gang Xu, Zhi-Wei Lin, Xiao-Yi Chen, Jin-Yang Chen, Xiao-Zhou Mou, Pei-Yang Hu

**Affiliations:** ^1^ Department of Vascular Surgery, Tiantai People’s Hospital of Zhejiang Province (Tiantai Branch of Zhejiang People’s Hospital), Taizhou, Zhejiang, China; ^2^ Department of Cardiovascular Medicine, Heart Center, Zhejiang Provincial People’s Hospital (Affiliated People’s Hospital), Hangzhou Medical College, Hangzhou, China; ^3^ Clinical Research Institute, Zhejiang Provincial People’s Hospital (Affiliated People’s Hospital), Hangzhou Medical College, Hangzhou, China; ^4^ Zhejiang Healthfuture Biomedicine Co., Ltd., Hangzhou, China; ^5^ Department of Traumatology, Tiantai People’s Hospital of Zhejiang Province (Tiantai Branch of Zhejiang People’s Hospital), Taizhou, China

**Keywords:** targeted drug delivery, exosome, cardiovascular disease, mesenchymal stem cells, extracellular vesicles

## Abstract

In recent years, stem cells and their secretomes, notably exosomes, have received considerable attention in biomedical applications. Exosomes are cellular secretomes used for intercellular communication. They perform the function of intercellular messengers by facilitating the transport of proteins, lipids, nucleic acids, and therapeutic substances. Their biocompatibility, minimal immunogenicity, targetability, stability, and engineerable characteristics have additionally led to their application as drug delivery vehicles. The therapeutic efficacy of exosomes can be improved through surface modification employing functional molecules, including aptamers, antibodies, and peptides. Given their potential as targeted delivery vehicles to enhance the efficiency of treatment while minimizing adverse effects, exosomes exhibit considerable promise. Stem cells are considered advantageous sources of exosomes due to their distinctive characteristics, including regenerative and self-renewal capabilities, which make them well-suited for transplantation into injured tissues, hence promoting tissue regeneration. However, there are notable obstacles that need to be addressed, including immune rejection and ethical problems. Exosomes produced from stem cells have been thoroughly studied as a cell-free strategy that avoids many of the difficulties involved with cell-based therapy for tissue regeneration and cancer treatment. This review provides an in-depth summary and analysis of the existing knowledge regarding exosomes, including their engineering and cardiovascular disease (CVD) treatment applications.

## 1 Introduction

Cardiovascular disease (CVD) and its complications are expected to account for a significant proportion of morbidity and death cases worldwide, with an estimated 23.6 million cases anticipated by the year 2030 (Clark, 2013; WHO, 2017). CVD encompasses a range of conditions, including coronary heart disease, deep vein thrombosis, pulmonary embolism, and myocardial infarction (MI). These conditions are characterized by the occurrence of ischemia and subsequent tissue death. MI and heart failure are the primary conditions that might result in mortality (Chandarana et al., 2018). Morphogenesis, cardiac rhythm, and muscle function and repair are all abnormalities associated with cardiac disease (Ouyang and Wei, 2021). Hypertension, atherosclerosis, and dyslipidemia are the principal etiological factors contributing to the prevalence of CVD. MI can result in a loss of approximately 25% of cardiomyocytes within the heart (Weinberger and Riley, 2023). Due to the inability of adult cardiomyocytes to proliferate, the heart is incapable of generating new cardiomyocytes to replace those that have been lost. It therefore self-heals through the formation of unbeatable fibrotic scar tissue. The gradual process of ventricular remodeling, commonly referred to as the healing process, leads to the weakening of the cardiac muscles. This weakening subsequently leads to a decline in contractility and muscular strength, ultimately resulting in the development of congestive heart failure and eventual death (Prajnamitra et al., 2019). One potential strategy for addressing heart disorders involves the targeted administration of cardioprotective medications directly to the infarcted myocardial and cardiovascular systems. Hence, the need for improved therapeutic interventions and the utilization of numerous drugs within the cardiac domain led to the emergence of targeted drug delivery. The knowledge, application, and implementation of nanoscience in medicine are novel methods for treating CVD and have the potential to enhance CVD treatment (Hu et al., 2020).

In numerous human tissues, mesenchymal stem cells (MSCs) are found and perform a variety of functions. They are present in a variety of tissues, including the placenta, adipose tissue, spinal cord, umbilical cord tissue, and blood (Kou et al., 2022). Due to its low immunogenicity, versatile differentiation capacity, and notable homing ability, it exhibits considerable promise for research in the fields of CVD, nervous system, and hematological disorders (Zhuang et al., 2022). Due to its advantageous properties, the MSC-based drug delivery system has emerged as a promising therapeutic option. Nevertheless, the use of cells as drug carriers presents several challenges, including unpredictable differentiation incidents, infection, cell embolism, storage, and production issues (Su et al., 2021). The exosomes, being the secretion of MSCs, possess the inherent benefits of MSCs while also addressing the limitations associated with MSCs as vehicles for drug delivery (Tang et al., 2021).

Exosomes, classified as extracellular vesicles (EVs), exhibit a size range of approximately 40–160 nm and are released by many eukaryotic cells. By fusing multivesicular bodies across the plasma membrane, these are generated, subsequently liberating their intracellular contents into the extracellular environment (Kalluri and LeBleu, 2020). Extensive study has been conducted on the properties of exosomes and their prospective applications as delivery vehicles for small molecule treatment (Liu et al., 2018a). Exosomes can be easily obtained from different tissues in the body and circulate freely in the bloodstream. Their contents have different impacts based on the type and health of the cell they come from. Given their distinct attributes and mechanisms, they are pivotal elements in controlling cardiovascular function and have a significant impact on nearly all aspects of cardiovascular pathology (Zheng et al., 2021a). Exosomes transport a diverse range of micro (mi)RNAs that directly impact several key factors of CVD, including angiogenesis, hypertrophy, apoptosis, damage, fibrosis, and repair. Exosomes generated from MSCs are vital to MSC-based treatment of CVDs, including as MI and reperfusion damage. In contrast to MSC transplantation, the administration of exosomes derived from MSCs has demonstrated therapeutic potential for CVDs, including the ability to prolong cardiomyocyte survival and prevent apoptosis. Consequently, exosomes derived from MSCs appear to be a viable strategy for the treatment of CVDs (Arslan et al., 2013). As a result of the active secretion of exosomes by numerous types of MSCs, they have garnered considerable interest in the context of CVD. They facilitate the transfer of cytoplasmic cargo across cells and have therapeutic and diagnostic applications (Hirai et al., 2020). An illustrative instance can be found in the study conducted by Mayourian et al., wherein they discovered that exosomes derived from human MSCs (hMSCs) affect cardiac contraction by mediating the excitation–contraction coupling of the heart via miR-21-5p, thereby regulating cardiac contraction (Mayourian et al., 2018). Furthermore, there has been growing interest in utilizing exosomes as a promising nanoscale platform for the targeted delivery of therapeutic drugs or genes. This approach holds great potential for targeted treatment of cardiac diseases, particularly when employing artificially enhanced exosomes with superior cardiac targeting capabilities (Wan et al., 2020). Additional techniques, such as the modification of polypeptides on exosomes, may also be employed to enhance the specificity of cardiac targeting. Engineered exosomes possess distinct features as natural nanoparticles (NPs) that distinguish them from other synthetic nanocarriers. This unique feature enables them to serve as a novel approach for the interventional treatment and early diagnosis of CVD. Specifically, it demonstrated efficacy in promoting the differentiation of cells, preventing apoptosis, and facilitating targeted modification for cardiac homing through their paracrine effect. These effects could inhibit inflammatory responses, mitigate fibrosis, and enhance heart homing, thereby offering promising therapeutic benefits for CVD. This review will examine the current developments, significant obstacles, and opportunities for the applicability of exosomes derived from MSCs. This review aims to provide valuable insights into the potential advancements in the design and development of exosomes derived from MSCs, as well as the prospects for their therapeutic application.

## 2 Biogenesis and functionality of exosome

**FIGURE 1:**
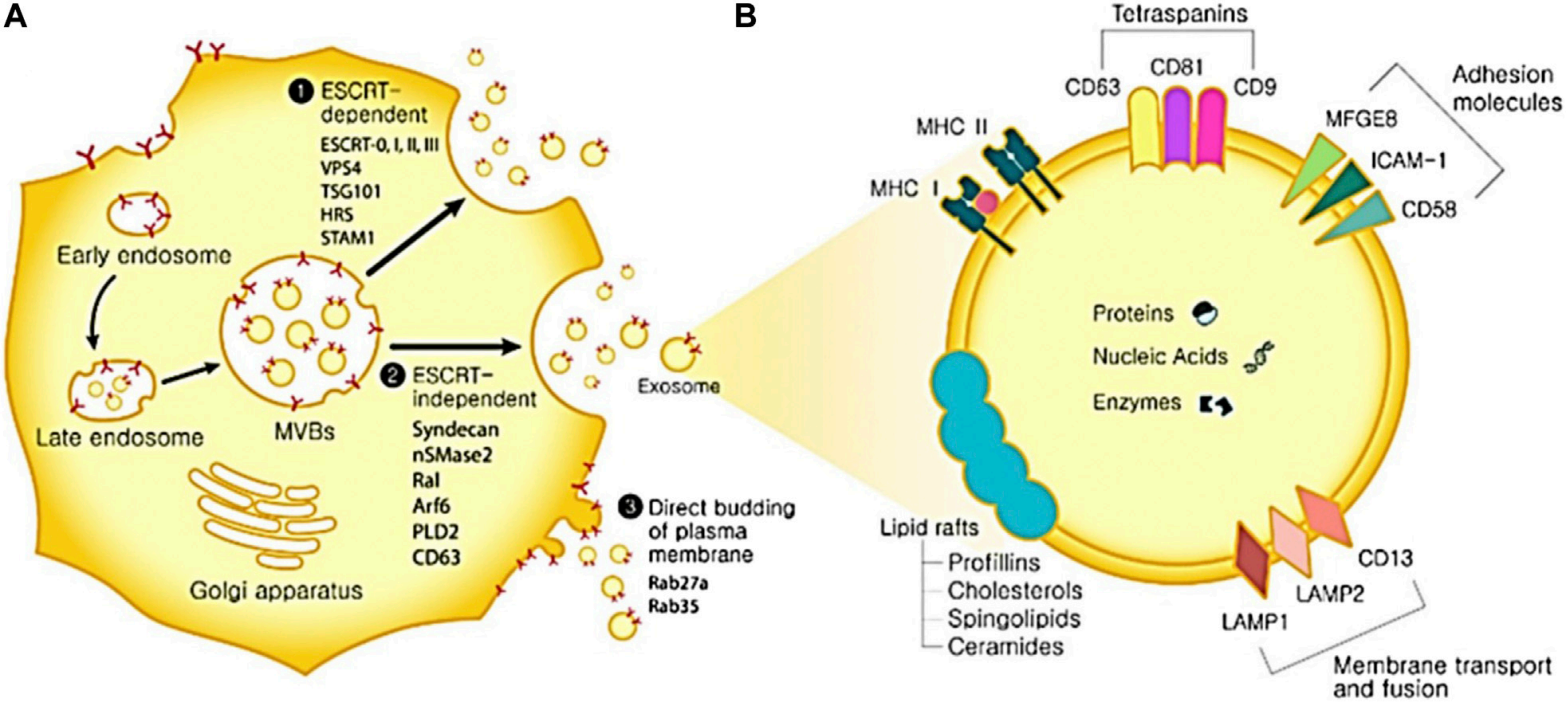
The biogenesis of exosomes and its uses. **(A)** Biogenesis of exosomes. Exosome biogenesis can occur via three distinct routes. ESCRT-dependent pathway and associated proteins. 2. ESCRT-independent pathway and associated proteins. 3. Direct budding of the plasma membrane and associated proteins. **(B)** Components of exosomes. ICAM-1: intercellular adhesion molecule 1; MFGE8: milk fat globule-EGF factor 8 protein; LAMP1,2: lysosomal-associated membrane protein 1,2; MAPK: mitogen-activated protein kinase; MHC I, II: major histocompatibility complex I, II. ERK: extracellular signal-regulated kinase; PGK1: phosphoglycerate kinase 1; GAPDH: glyceraldehyde 3-phosphate dehydrogenase.

EVs are classified based on their size, specifically their diameter. These categories include apoptotic bodies, which have a diameter greater than 1,000 nm; microvesicles, ranging from 100 to 1,000 nm in diameter; and exosomes, with a diameter between 30 and 150 nm (Gurunathan et al., 2019). They were discovered in a variety of physiological fluids, including milk, saliva, urine, blood, and amniotic fluid. It is now recognized that EVs play a role in pathological and physiological processes by mediating intracellular communication (Minciacchi et al., 2015). As therapeutic nanocarriers and non-invasive diagnostic biomarkers for the treatment of numerous diseases, including cancer, cardiovascular dysfunction, and neurodegeneration, EVs exhibit potential in a number of medical applications (Zhang et al., 2019a). Exosomes are generated via the process of endosomal membrane inward budding, leading to the development of ILVs, or intraluminal vesicles. These ILVs subsequently undergo conversion into multivesicular bodies (MVBs) Figure 1 (Yuan et al., 2022). The fusion process encompasses the participation of multiple Rab GTPases, while the discharge of ILVs into the endosomal lumen is facilitated by the Endosomal Sorting Complexes Required for Transport (ESCRT) machinery (Dixson et al., 2023). Endosomal membrane ubiquitinated proteins are recognized and sequestered by ESCRT 0, which in turn recruits ESCRT I and II. Ubiquitin functions as a signaling molecule that facilitates the transportation of membrane proteins or impaired cellular constituents to the lysosome for disintegration. Additionally, it acts as a signal for the sorting of exosomal cargo on the endosome membrane (Zhang et al., 2021). MVB is capable of fusing with either lysosomes for deterioration or the plasma membrane to liberate exosomes. The initiation of intraluminal membrane budding is facilitated by the binding of ESCRT I and II to the endosomal membrane’s outer surface near the ubiquitinated protein cargos. This binding process selectively targets these cargos to be included within the newly-formed intraluminal buds in the MVB. The procedure is completed by ESCRT III sequestering MVB proteins. Nevertheless, inhibiting the ESCRT pathway did not prevent MVB production, indicating the existence of an ESCRT-independent process. When ALIX and syntenin co-accumulate with exosomes, ubiquitous transmembrane proteins called syndecans directly control ILVs during exosome production (Li et al., 2022a). By encouraging MVB budding and enriching exosomes with CD63, proteolipoprotein, TSG101, and CD81, neutral sphingomyelinase (nSMase) promotes the production of ILVs.

A lipid bilayer membrane encapsulates exosomes, which are composed of ceramide, sphingomyelin, and cholesterol. Certain lipid constituents, such as prostaglandins and phosphatidylserine, might be involved in exosome function (Qiao et al., 2023). The tetraspanin family, including CD63, CD9, and CD81 represents the prevailing proteins that function as markers on the surface of exosomes (Zhu et al., 2023). Heat shock proteins, membrane transporters, fusion proteins, lipid-related proteins, phospholipases, and MVB biogenesis proteins are among the other frequent proteins (Gupta et al., 2022). Exosomes are known to encompass microRNAs (miRNAs) and mRNAs. Endocytosis of exosomes by recipient cells can induce protein expression changes in those cells due to the genetic information conveyed by the RNAs (Li et al., 2023a). Exosomes, a cellular entity, possess the potential for application in disease detection, drug administration, and therapeutic interventions owing to their distinctive bioactive compounds. Non-invasive isolation of these biomarkers from patients renders them promising candidates for diagnostic purposes in several diseases, including cancer, metabolic conditions, neurodegenerative, and infectious diseases (Li et al., 2022b; Motallebnezhad et al., 2022). Exosomes also have potential drug delivery features, such as the ability to penetrate the blood-brain barrier and resist degradation by RNase activity during migration (Li et al., 2022b). Exosomes possess a structural resemblance to liposomes, hence affording protection to their payload against the external environment. In addition, they may serve as a viable substitute for cell treatment due to their enhanced safety and controllability relative to therapeutic approaches employing live cells (Ragni et al., 2022). In contrast to cells, exosomes do not undergo mutation, replication, or promote metastasis. However, it is essential to conduct careful verification of the correlation that exists between the biomarker and the disease.

## 3 Isolation of exosomes

**FIGURE 2:**
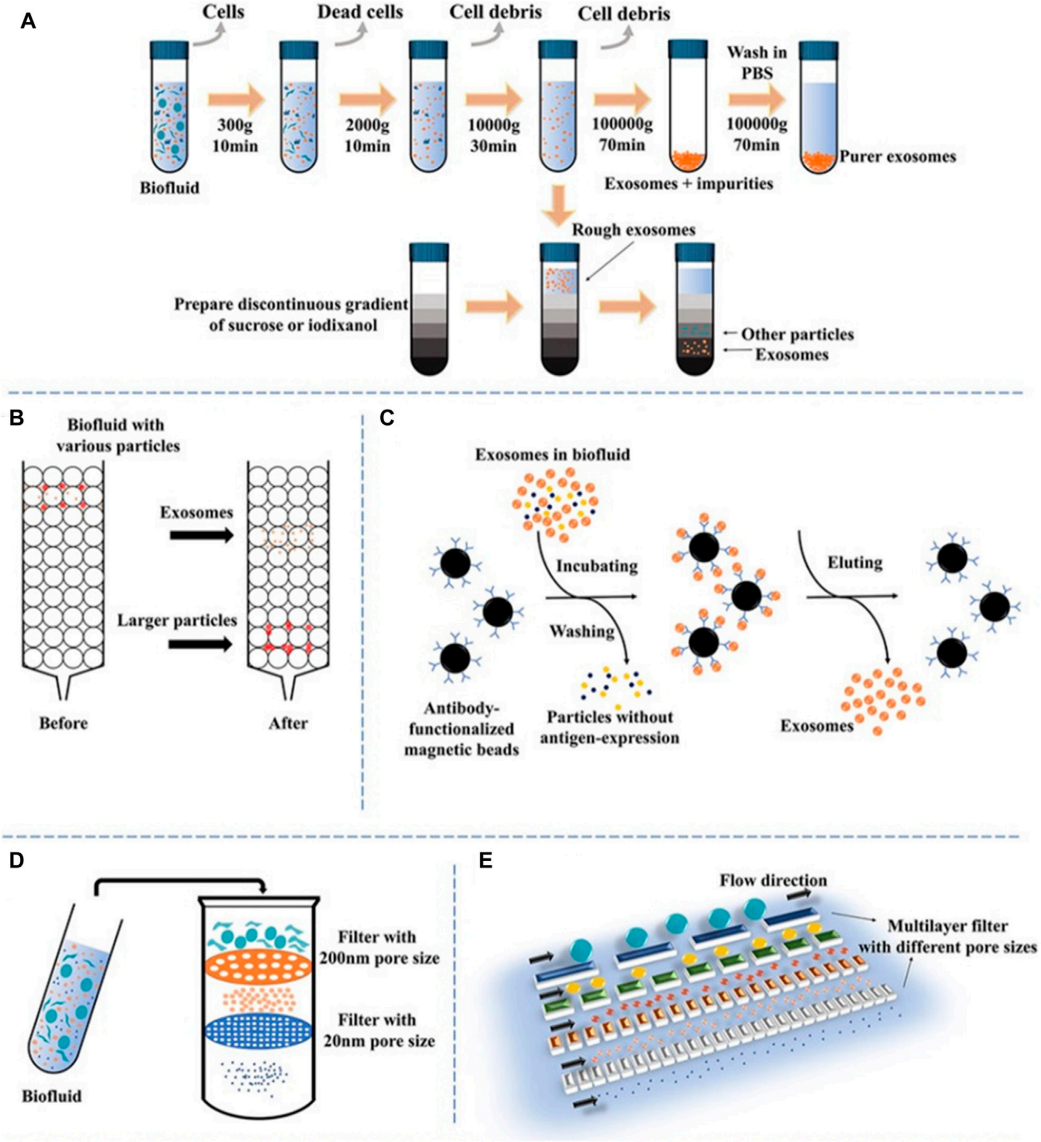
A schematic illustration of prevalent techniques utilized in exosomal separation. **(A)** Ultracentrifugation and density gradient centrifugation, **(B)** size-exclusion chromatography, **(C)** immunoaffinity, **(D)** dead-end filtration (DEF), and **(E)** microfluidic flow filtration (Chen et al., 2022).

The accurate isolation of exosomes is of utmost importance in the assessment of their biological functions. Nevertheless, the isolation of exosomes can provide challenges due to their diverse content, varying sizes, many sources, and distinct functions Figure 2 (Chen et al., 2022). Numerous current isolation techniques exhibit limitations in achieving complete separation of exosomes and extracellular vesicles, as well as lipoproteins originating from nonendosomal pathways, due to their comparable physicochemical characteristics (Nguyen et al., 2019). Consequently, the purity of exosomes may be diminished. The efficient enrichment of exosomes holds significant importance, prompting the selection of diverse isolation methods designed for specific applications and objectives. The following methods are frequently employed: polymer precipitation, size-based isolation, immunoaffinity capture, and ultracentrifugation (Li et al., 2017; Luo et al., 2018a).

### 3.1 Ultracentrifugation

Ultracentrifugation (UC) is a commonly employed technique in the field of exosome extraction, recognized for its superior efficacy in separating and extracting exosomes from other constituents by exploiting disparities in density and size (Shirejini and Inci, 2022). The procedure entails a two-step approach: initial centrifugation at a low to moderate speed to eliminate undesired constituents, followed by a subsequent high-speed extraction of exosomes at 100,000 × g. Time, rotor type, and centrifugal force are variables that can influence the yield and purity of exosomes. To eliminate impurities, exosomes are frequently rinsed with PBS (Théry et al., 2006). To enhance the purity of exosomes, density gradient centrifugation is frequently employed in conjunction with ultracentrifugation. As a result of the elevated viscosity of the sucrose solutions utilized in this method, sedimentation time may be prolonged (Cvjetkovic et al., 2014). High-purity separation of exosomes from cells infected with human immunodeficiency virus has been accomplished using an alternative technique, iodixanol gradient centrifugation (Zhou et al., 2020a). Cantin et al. (2008) discovered distinct sedimentation rates in the iodixanol gradient, which allowed them to successfully separate exosomes from infected cells. This approach made it easier to collect exosomes with high purity. Although density gradient centrifugation offers the benefit of enhancing exosome purity, its utilization of sucrose solutions with a high viscosity results in a decrease in the sedimentation rate of exosomes, thus extending the duration of the procedure (Ford et al., 1994).

### 3.2 Ultrafiltration

The ultrafiltration technique relies on the utilization of a membrane possessing a specified molecular weight cutoff (MWCO) or certain pore diameter to segregate particles based on their size, hence serving as a size-dependent isolation methodology (Kurian et al., 2021). Devices for ultrafiltration can be divided into two categories: tandem-structured and sequential. The kind of filter utilized has an impact on the exosome recovery efficacy. In one investigation, for example, cellulose membranes with pore sizes of 10 kDa had the best rescue performance (Vergauwen et al., 2017). In addition to significantly reducing processing time, ultrafiltration does not necessitate the use of specialized apparatus. However, membrane fouling poses a substantial challenge, resulting in diminished operational efficacy and a reduced lifespan of membranes (Chang et al., 2022). Tangential flow filtration (TFF) approaches can provide a viable option through the implementation of cross-flow filtration. The tangential flow component significantly enhances the permeability flow by preventing the development of the concentration polarization layer. By reducing the possibility of impurities and exhibiting higher consistency than traditional isolation techniques, TFF lowers the possibility of pore blockage (Haraszti et al., 2018). Nevertheless, a drawback of ultrafiltration is the presence of NPs that are similar in size to exosomes, which might result in the clogging of pores. Combining ultrafiltration with alternative techniques may efficiently reduce this issue (Abiri et al., 2019).

### 3.3 Polymer precipitation

The polymer precipitation approach is commonly employed in exosome extraction and purification. It operates on the principle of decreasing the solubility of exosomes by applying the application of particular centrifugation conditions, utilizing poly (ethylene glycol) as the medium (Oh et al., 1988). Because viruses and exosomes share comparable biophysical characteristics, this technique, which was initially developed for virus separation, can be applied to exosomes as well (Le and Fan, 2021). The polymer precipitation approach offers numerous notable advantages, such as its capacity to effectively process substantial sample volumes, its user-friendly operational characteristics, and its ability to yield prompt analysis results. However, the recovery rate and purity of exosomes may be comparatively low, and the existence of residual polymers may cause false positive results and disrupt subsequent functional analysis. To overcome these constraints, other purification techniques, including size exclusion chromatography (SEC) or ultracentrifugation, can be utilized to enhance the purity of exosomes and eliminate any remaining impurities (Diaz et al., 2018).

### 3.4 Size-based isolation

To isolate and purify exosomes, a common strategy is to combine SEC with ultrafiltration. SEC depends on the variations in size between exosomes and other components in biological samples (Benedikter et al., 2017). The approach employs the separation of macromolecules according to their ability to enter pores, whereas tiny molecules are separated by the use of a mobile phase. Easy, quick, and affordable methods for isolating exosomes can be achieved with commercially accessible columns based on SEC like qEV separation columns, EVs second purification columns, and Exospin exosome purification columns. The integrity of isolated exosomes is, nevertheless, compromised by the method’s susceptibility to contamination by particles of comparable size (Böing et al., 2014). In contrast, samples are selectively separated via ultrafiltration using membranes with varying MWCO. This technique is extensively employed in the field of exosome research due to its cost-effectiveness, notable enrichment efficacy, and capacity to maintain exosome function. Nonetheless, nonspecific membrane binding to exosomes is an additional drawback of ultrafiltration, which contributes to diminished recovery rates and poor purity. Ultrafiltration and SEC have been utilized in tandem to isolate exosomes of exceptional purity. This approach presents enhanced exosome purity, and its favorable recovery rate and ability to maintain exosome activity render it a compelling choice for numerous exosome investigations (Xu et al., 2023).

### 3.5 Immunoaffinity chromatography

The immunoaffinity technique is a specialized and extremely sensitive approach for purifying and separating substances, relying on the particular binding interactions between ligands and antibodies (Chen et al., 2019). The approach is predicated on the distinct identification of surface biomarkers present on exosomes, including proteins associated with the ESCRT complex. These biomarkers should be prevalent on the surfaces of exosome membranes. Similar results to ultracentrifugation can be obtained with immunoaffinity chromatography but at a lower volume. Additionally, it is capable of being employed for both quantitative and qualitative exosome detection. The technique offers several benefits, including excellent specificity, high sensitivity, high yield, and high purity (Tschuschke et al., 2020). Enzyme-linked immunosorbent assay (ELISA) approaches utilizing microbes can be employed to facilitate the enrichment of various body fluids, such as serum, plasma, and urine. The efficiency of exosomes is comparable to ultracentrifugation, according to quantitative analysis using immunoaffinity chromatography, however, a significantly smaller sample is needed (Zarovni et al., 2015). Furthermore, exosomes can be purified in a highly specific manner using immunoaffinity chromatography, which does not necessitate the use of specialized apparatus or extended processing times. Nevertheless, the storage of exosomes obtained using immunoaffinity chromatography has certain challenges, and this technique may not be optimal for the extraction of exosomes on a large scale (Theel and Schwaminger, 2022).

### 3.6 Microfluidics-based isolation

Microfluidic devices have been recognized as a high-throughput approach for the isolation of exosomes, employing principles including size density and density. The immune-microfluidic method is widely employed as the predominant microfluidic technology, wherein antibodies are selectively bound to immobilized exosome markers on microfluidic chips (Chen et al., 2010). The ExoChip, a microfluidic device commonly employed for exosome isolation, utilizes CD63 antibodies. Additional microfluidic systems that can be employed in various applications encompass gold electrodes with CD9 antibodies, CD81 antibodies with nanointerfaces made of graphene oxide and polydopamine, and CD9 antibodies in herringbone grooves (Zhang et al., 2016). This method has the benefit of isolating exosomes quickly and efficiently while maintaining a high yield. Despite the complexity and expense of microfluidic devices, they are more economical than immunoaffinity capture (Carnino et al., 2019). Depending on the device type and the duration of the flow channel, exosomes may be isolated from sample volumes. However, this approach necessitates the use of specialized equipment and has not yet gained widespread acceptance as a standardized technique for exosome isolation (Contreras-Naranjo et al., 2017). Despite these constraints, microfluidic devices have demonstrated significant promise in extracting exosomes, and further advancements in this domain could facilitate the widespread implementation of this technique (Contreras-Naranjo et al., 2017).

## 4 Surface modification of exosomes for cardiovascular targeting

Deriving and target cells exhibit distinct mechanisms of exosome binding and uptake. Target cells internalize them by phagocytosis, endocytosis, micropinocytosis, and membrane fusion. The surface molecules, including tetraspanins, proteoglycans, integrins, and lectins, are of utmost importance in facilitating these activities (He et al., 2018). Surface modification is the primary method employed to impart myocardial-targeting specificity to exosomes, given the established evidence of differential protein expression between ischemic and normal myocardium (Thupakula et al., 2022). The targeting performance of exosomes has been enhanced by engineering them to over-express surface peptides with ischemic myocardial selectivity. Numerous myocardial homing peptides have been discovered and validated using *in vivo* biopanning and phage display methodologies, which are commonly employed for the detection of cell-targeting peptides (CTP). These peptides exhibit the ability to specifically target ischemic myocardium (Wang et al., 2021a).

**FIGURE 3:**
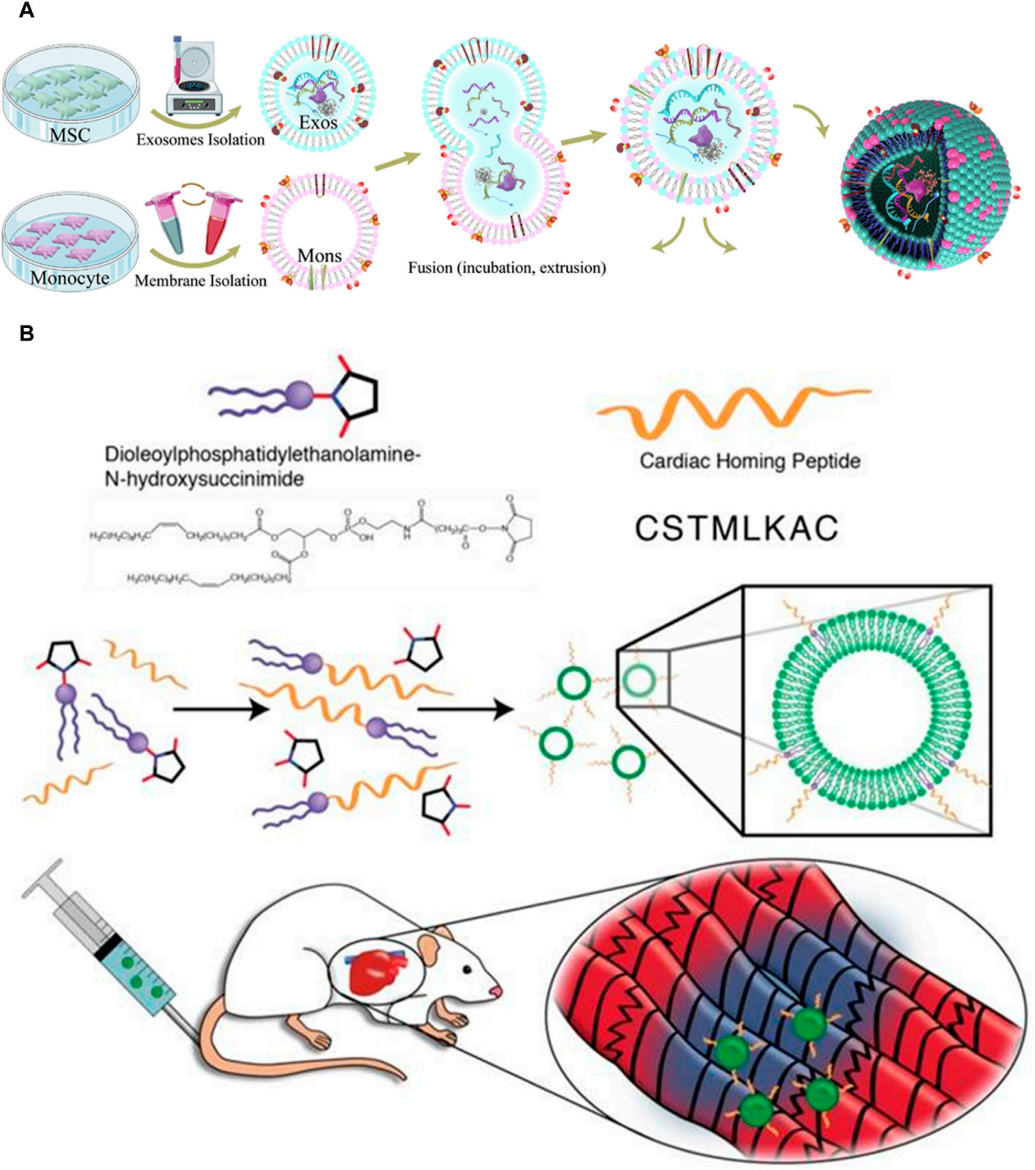
**(A)** Exosome isolation and fusion with monocytes (Mons) through serial extrusion to enhance endothelial development during angiogenesis and regulate macrophage subpopulations following MI (Zhang et al., 2020). **(B)** The reaction of DOPE-NHS with cardiac homing peptide (CHP) produced exosomes that target the myocardium. The lipophilic ends of the DOPE-CHP compound have an amazing ability to spontaneously insert themselves into the exosomal membrane, covering the exosome with a layer of protection made up of CHP peptide (Vandergriff et al., 2018).

Surface modification can be accomplished using several techniques, including biological, chemical, and physical processes. In the context of biological modification, gene transfer vectors, such as lentiviruses or adeno-associated viruses (AAVs), can be employed to introduce the genetic sequences encoding a myocardium-targeting peptide into the genome of exosome-donor cells. This genetic modification enables the host cells to express a peptide that facilitates homing to the myocardium. The myocardial homing peptide is subsequently combined with exosome surface-expressed transmembrane proteins (Witwer and Wolfram, 2021). The lysosome-associated membrane glycoprotein 2B (LAMP-2B) is a member of the lysosomal-associated membrane protein (LAMP) family and is prominently expressed on the membrane of EVs. It is widely utilized as a membrane protein in various applications (Salunkhe et al., 2020). The protein consists of two distinct regions: an extramembrane domain located at the N-terminus and a transmembrane domain located at the C-terminus. To generate a specific targeting effect, the targeting peptide can be combined with the extracellular domain of LAMP-2B at the N-terminus. Cardiac-targeting peptide (CTP)-Lamp2B vectors were introduced into HEK 293 cells by Kim et al. to produce CTP-expressing EVs (Kim et al., 2018). The efficiency at which CTP-expressing EVs are distributed to the heart was enhanced in both *in vitro* and *in vivo* settings. In contrast to conventional EVs, the EVs engineered with myocardium-targeting peptides showed a notable increase in their ability to specifically target ischemic myocardium. This led to a noteworthy reduction of cardiomyocyte apoptosis and inflammation, as well as an enhancement in angiogenesis and a reduction in infarct size in a mouse model of MI (Figure 3A). Mentkowski et al. utilized a comparable methodology in their study, wherein they genetically modified cardio-sphere-derived cells (CDCs) to incorporate the expression of Lamp2b. This was subsequently fused with a peptide specific to cardiomyocytes, namely, WLSEAGPVVTVRALRGTGSW (Mentkowski and Lang, 2019). The EVs extracted from the aforementioned on the surface led to an increased cardiac retention.

Chemical modifications can be applied to the surface of exosomes after their isolation. In contrast to the biological modification approach, chemical reactions exhibit greater speed, efficiency, and simplicity. In their study, Zhu et al. employed bio-orthogonal chemistry to conjugate exosomes with a peptide that specifically targets the ischemic myocardium. The researchers observed that the modified exosomes exhibited a notable affinity for the ischemia lesions in the injured heart, leading to a significant cardioprotective effect during MI (Zhu et al., 2018). In a previous study, Antes et al. introduced a novel method called “cloaking” to effectively incorporate targeting molecules onto the surfaces of exosomes through a membrane anchoring platform (Antes et al., 2018). The cloaking system is made up of three different components: a polyethylene glycol spacer, A conjugated platform molecule of streptavidin, and a phospholipid membrane anchor made of DMPE. Any biotinylated molecule can be attached to the platform molecule to decorate exosomes. By conjugating exosomes with cardiac homing peptide via a dioleoylphosphatidylethanolamine N-hydroxysuccinimide (DOPE-NHS) linker, Vandergriff et al. showed enhanced therapeutic results and increased homing efficacy of the injured heart Figure 3B (Vandergriff et al., 2018).

Membrane fusion is a physical method of modifying the surface of exosomes, as opposed to chemical or genetic methods. Zhang et al. successfully produced monocyte mimetic exosomes by fusing MSC-derived exosomes with monocyte cell membranes via the fusion-extrusion technique (Zhang et al., 2020). These modified exosomes possessed the inflammatory targeting characteristics of monocytes, and they were able to penetrate damaged myocardium through the bloodstream. The aforementioned technique demonstrated notable efficacy in selectively targeting damaged myocardial tissue, resulting in enhanced therapeutic effects on heart function. Hu et al. combined MSC-derived exosomes and platelet membranes by extruding them via 200 nm filters, which improved their capacity to target injured tissue and enhanced the internalization of cells through macropinocytosis (Zhang et al., 2020). By modifying the exosomes with platelet membranes, they were able to increase their uptake by cardiomyocytes and endothelial cells while decreasing their uptake by the mononuclear phagocyte system *in vivo*.

Exosomes were endowed with enhanced targeting capabilities through surface peptide engineering. The effects of these technologies, however, on the biological characteristics of exosomes remain unknown. Additional research is required to determine whether the surface engineering performance may have undesirable effects, including immunogenic activities, or alter the typical functions of proteins in the exosome membrane. Modifying the cells that produce exosomes is a laborious and time-consuming process, and these methods have the potential to compromise the integrity and stability of the exosome membrane or result in a poor exosome yield (Lee et al., 2016; Armstrong et al., 2017). Furthermore, surface modification is incapable of simultaneously loading multiple surface peptides. Peptides exposed on the surface of exosomes are also unstable and susceptible to degradation (Lee et al., 2016). Hung et al. provided evidence that the fusion of LAMP-2B with membrane display could potentially result in the loss of ligands (Pessentheiner et al., 2013). Glycosylation motif–containing designed targeted peptide–Lamp2b fusion proteins could be useful in protecting peptides from degradation.

## 5 Engineered exosomes as biocarriers

Exosomes have been utilized as biocarriers for the delivery of various therapeutic agents, including macromolecules, noncoding RNA (ncRNAs), and bioactive proteins in the context of CVD treatment. The biological origins of exosomes, including endothelial cells, fibroblasts, and smooth muscle cells, play an essential role in the cardiac system. Exosomes have favorable characteristics such as low biocompatibility and immunogenicity, rendering them highly promising as drug carriers in the field of medicine and therapeutic interventions (Liu et al., 2021a). The heterogeneity of natural exosomes restricts their applicability in CVD. Immunosuppressive, immunomodulatory, and regenerative effects of exosomes are improved by preconditioning other cells or stem cells triggered by chemicals, hypoxia, and cytokines (Zhang et al., 2019b). Tumor necrosis factor, deferoxamine, nitric oxide, lipopolysaccharides (LPS), and interferon increase the effectiveness of exosomes. Similar improvements were observed when cytokines, including hypoxia-inducible factors (HIF) and interleukins (ILs), were stimulated (Nocera et al., 2019). The effectiveness of exosomes is further enhanced by ultraviolet induction and miRNAs (Hegyesi et al., 2019). The therapeutic efficiency of exosomes is enhanced through stem cell surface modification and gene transfection (Bu et al., 2021).

### 5.1 Stimulated exosomes

To increase the therapeutic efficacy of exosomes in cardiac protection, several methods have been developed to enhance their activity. Different methods, including exercise, medications, low oxygen conditions, and others, are employed to induce exosome-mediated beneficial effects that have the potential to ameliorate CVD. The immunomodulatory, immunosuppressive, and regenerative effects of stem cells or other cells activated with HIF, cytokines (ILs), chemicals, and hypoxia can be enhanced through preconditioning (Zheng et al., 2021b). The effectiveness of specific exosomes is enhanced by LPS, deferoxamine, nitric oxide, tumor necrosis factor, and interferon (Nocera et al., 2019).

EVs and microbubbles (MBs) are nanoparticles in drug-delivery systems that are both considered important for clinical translation. Current research has found that both MBs and EVs have the potential to be utilized as drug-delivery agents for therapeutic targets in various diseases (Ullah et al., 2021). MBs have a gaseous core (perfluorocarbon gas) with a protein, polymer, or phospholipid coating (Aliabouzar et al., 2016). Acoustic pressure expands and contracts MBs in an ultrasound (US) field due to their compressible internal gas phase. Oscillation under US, internal gas leakage due to shear stress by US mechanical force, and cavitation by intense ultrasound can dissipate MBs. US-induced MB cavitation causes jet-stream and produces temporary pores on the surrounding cell membrane. Therapeutic substances, including genetic material and chemical drugs, can be efficiently transported via the transient formed pores (Jang et al., 2023). MBs may be more easily disrupted at the US site, which might lead to an increase in cell membrane permeability and recipient cell absorption. An effective method to enhance the efficiency of exosomes is by utilizing ultrasound-targeted microbubble disruption (UTMD), which facilitates the delivery of exosomes into adipose tissue. When stimulated with LPS, bone marrow MSC exosomes prevent nuclear factor-κB from being transported to the nucleus (Villarreal-Leal et al., 2021). Immunomodulatory effects are enhanced by improved cardiac function and protection against apoptosis. According to a study, prolonged exercise has been found to induce the activity of plasma exosomal miR-342-5p, which serves as a carrier for promoting heart protection (Hou et al., 2019). Additionally, hypoxia preconditioning is an efficient stimulation technique. hMSCs preconditioned to hypoxia can provide exosomes with cardioprotective properties; in this regard, miRNA-26a was utilized to downregulate the expression of GSK3β in a model of ischemia-reperfusion injury. After MI, loading these exosomes into a hydrogel prolonged their therapeutic effects and prolonged their typically short retention time (Kajimoto et al., 2013).

Atherosclerosis (AS) is a chronic inflammatory disease with a complex etiology that serves as the primary cause of CVD (Chen et al., 2017a). The main pathogenic features of AS include lipid accumulation, inflammatory cell infiltration, cell proliferation, and apoptosis. These conditions might eventually result in stroke and MI (Wang et al., 2019). As a result, it is critical to investigate the pathogenesis of AS and to designate molecules that have a close link with its occurrence and progression, as such efforts can have a substantial effect on the early detection and treatment of the disease, thereby preventing adverse events. Recent research studies have discovered that exosomes play a crucial role in inter-cellular communication by releasing inflammatory cytokines and triggering excessive inflammatory responses. This suggests that exosomes are closely associated with the onset and progression of cardiovascular disease, such as AS (Hulsmans and Holvoet, 2013). In conjunction with their contents, exosomes can serve as an indicator for the diagnosis and prognosis of AS, and sometimes as a vehicle for targeted therapy. Many studies indicate that anti-inflammatory therapy provides a novel avenue for the treatment of AS. IL-10 has a significant function in restricting inflammation and protecting against tissue damage. AS has been demonstrated to be inhibited by therapeutic delivery of IL-10 (Pinderski Oslund et al., 1999). In a study, the substitution of miR-122 with miR-155 in inflammatory atherosclerosis led to the activation of IL-10 mRNA, thus triggering the inflammatory response. miR-155 stimulates the expression of IL-10 mRNA in inflammatory macrophages. Engineered IL-10 mRNA can be passively encapsulated into exosomes through forced expression in donor cells, and subsequently transported to macrophages and specific other cell types in ApoE (−/−) mice plaques. The delivery of IL-10 via engineered exosomes, which decreases atherosclerosis in ApoE (−/−) mice, is an encouraging strategy (Liang et al., 2020; Bu et al., 2021). Additionally, MSC-derived exosomes are pivotal in AS pathogenesis and therapy. As an example, MSC-derived exosomal miR-21a-5p and miR-let7 have the ability to reduce AS, increase M2 reparative polarization, and decrease macrophage infiltration (Li et al., 2019; Ma et al., 2021). Similarly, endothelial cells can be protected against atherosclerotic damage by exosomes produced from adipose tissue-derived MSCs (ADSCs) containing miR-342-5p. Exosomal miR-100-5p, isolated from human umbilical cord MSCs, reduces AS by inhibiting eosinophilic granulocyte inflammation via the FZD5/Wnt/b-catenin pathway, according to the research (Xing et al., 2020; Gao et al., 2021). In brief, exosomes have the potential to be utilized for both AS diagnosis and treatment. The primary components of exosomes are protein and RNA, particularly exosome miRNAs, which have the ability to control target cell gene expression and physiological state, potentially playing a major role in the intercellular interference of CVD.

### 5.2 Hybrid exosomes

Exosome-liposome hybrid NPs serve as a vehicle for targeted drug delivery. These spherical NPs are used to load siRNA, or short interfering RNA. They carry several indicators and provide targeted siRNA to the appropriate cells. This particular hybrid possesses the capability to combine the benefits of both biological and synthetic gene delivery platforms, hence serving as novel carriers for siRNA (Evers et al., 2022).

Antibody-conjugated magnetic NPs can be delivered via exosomes for the treatment of MI. In infarcted heart tissue, novel NPs with a core of Fe_3_O_4_ and a shell of SiO_2_ bound to an antibody such as CD63 or MLC may interact with circulating exosomes. The MLC and CD63 antibodies have been identified as valuable markers that exhibit affinity for the surface of exosomes and exhibit accumulation within infarcted tissue. By reducing the infarct area and enhancing cardiac function, this method may offer a novel approach to the treatment of MI (Liu et al., 2020a). Exosomes are a promising carrier for the transport of biomolecules. A core-shell NPs system was developed, consisting of a Fe_3_O_4_ core and a silica shell. The particles were synthesized utilizing conjugation established through hydrazone bonds. Two antibodies have a high affinity for surface markers on injured cardiac cells, or towards the CD63 antigens located on the surface of exosomes. In rat and rabbit models of MI, exosomes expressing CD63 that are specific to infarcted tissue were captured by magnets. This capture process has the potential to modify angiogenesis and enhance the left ventricular ejection fraction.

**FIGURE 4:**
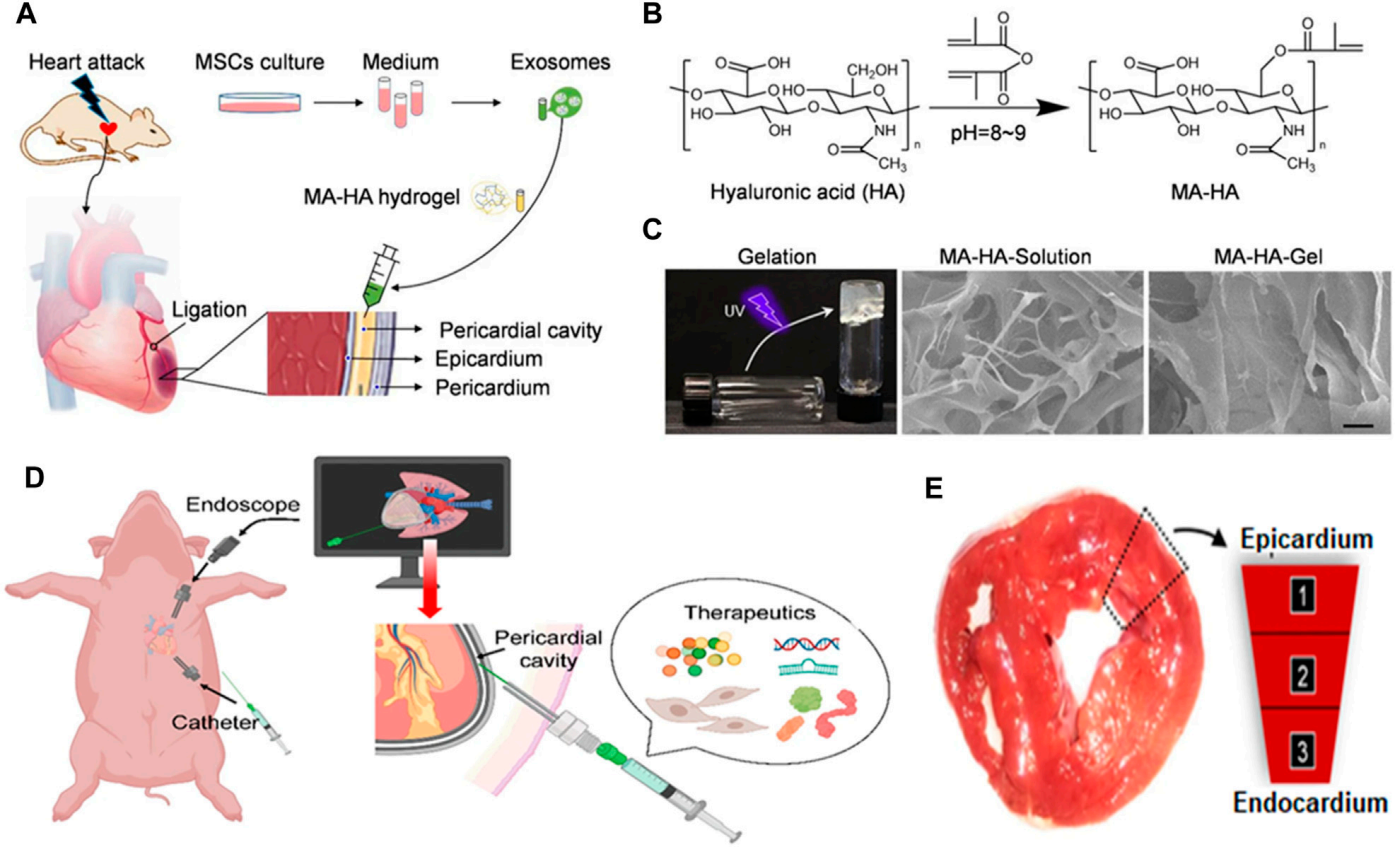
**(A)** Schematic illustrating exosome delivery intrapericardially for MI therapy and **(B)** development of an MA-HA hydrogel. **(C)** SEM images of MA-HA hydrogel prior to and subsequent to *in vitro* gelation induced by UV irradiation. **(D)** Schematic representation of therapeutics being delivered minimally invasively into the pericardial cavity of a pig using an endoscope. **(E)** Confocal microscopy images demonstrating cardiomyocyte assimilation of exosomes 3 days following iPC injection (Zhu et al., 2021).

Various methodologies have been employed to administer therapeutic agents to the cardiac region, employing either synthetic or natural injectable hydrogels, to address cardiac diseases. The limited cardiac retention hinders the potential utilization of intravenous administration. Following several modifications, exosomes have been engineered to serve as efficient vehicles for drug delivery in the development of drugs for cardiac repair. Additionally, they can be utilized as tissue engineering strategies to enhance their targeted distribution within the cardiac tissue (Huang et al., 2021; Pu et al., 2021). The hydrogel formulation has received extensive attention due to its notable characteristics, including excellent permeability, favorable biocompatibility, and biodegradability. The gelation duration of this substance is easily controllable, and it can replicate the structure and functionality of the extracellular matrix. This matrix plays a crucial role in cardiovascular repair and the therapy of cardiac ischemia (Yao et al., 2021). A delivery platform with less invasive characteristics was employed to administer therapeutic substances into the pericardial cavity using hydrogel injection, to facilitate heart repair (Yao et al., 2021). Upon administration, this innovative hydrogel assumed a comparable structure to a cardiac patch; thus, it represents a promising strategy for the delivery of therapeutic components in the context of cardiac therapy (Figure 4). Minimally invasive exosome sprays, which resemble cardiac patches, were used to administer hydrogels containing various stem cell-derived exosomes in another investigation. After MI, an innovative minimally invasive exosome spray was discovered to repair the damaged heart (Zhu et al., 2021). To address the issue of inefficient drug delivery to the heart and cardiac function, biomaterial-based exosome formulations were developed. This approach enhanced endogenous angiogenesis and decreased fibrosis in the heart following injury (Zhu et al., 2021). The role of MSC exosomes as paracrine factors in stem cells for heart regeneration has been identified. A functional peptide hydrogel was utilized to encapsulate human umbilical MSC exosomes for cardiac repair. Additionally, a three-dimensional hydrogel composed of hyaluronic acid was employed to encapsulate exosomes obtained from human cardiomyocytes (Derkus, 2021). Furthermore, an injectable hydrogel has been developed to repair cardiac injuries.

### 5.3 Delivery of biologically therapeutic protein

Various modification and pretreatment techniques, including electroporation, incubation, gene transfection, sonication, extrusion, hypotonic dialysis, and thermal shock, have been employed as effective strategies for the encapsulation of drugs, genes, and proteins into exosomes (Xi et al., 2021). These techniques are advantageous in inducing a cardioprotective impact on the modified exosomes. H3K27 demethylase UTX expression in mouse myocardial cells is decreased by the use of novel exosomes isolated from the Chinese patent drug Suxiao Jiuxin in conjunction with cardiac MSC therapy (Ruan et al., 2018a; Ruan et al., 2018b). After incubating HIF-1 with cardiac progenitors, Ong et al. indicated that external bodies might be used as drug vectors to treat MI (Ong et al., 2014). Overexpressed HIF-1 expressing cells release miR-126 and miR-210-rich exosomes. These miRNAs protect against myocardial ischemia. These exosomes stimulate miRNAs in mouse cardiomyocytes upon intravenous injection, increasing the myocytes’ resistance to hypoxia and decreasing apoptosis, ultimately aiding in the restoration of cardiac function. These techniques control the endogenous biodistribution of exosome treatment for CVD.

## 6 Applications of exosomes in CVD treatment

Stem cells-derived exosomes possess a wide range of functional RNAs, therefore presenting the potential for utilizing exosomes in facilitating the differentiation process of endothelial progenitor cells into smooth muscle cells, vascular endothelium cells, and cardiomyocytes within the damaged myocardium (Ringuette Goulet et al., 2018). Through the delivery of mRNA and miRNA, exosomes produced from MSCs, ESCs, EPCs, ADSCs, and iPSCs can affect cardiac protection (He et al., 2020).

### 6.1 Acute coronary syndrome (ACS)

ACS occurs when unstable coronary plaques erode or rupture, causing an acute myocardial infarction (AMI) (Zheng et al., 2021c). AMI and myocardial ischemia are treated with exosomes generated from a variety of cell sources, including EPCs, MSCs, iPSCs, and embryonic stem cells. Ischemia–reperfusion injury of the myocardium can result in arrhythmia, microvascular obstruction, and stunning, all of which have a profound impact on cardiac function and prognosis. Li et al. found that tanshinone IIA upregulates miR-223-5p, which in turn increases the therapeutic effect of MSC-derived exosomes in the HUVEC and H9C2 models (Li et al., 2023b). In addition, BMSC exosomes prevent damage caused by ischemia-reperfusion via altering autophagy, and pretreating H9C2 cell lines with oridonin enhanced the protective effect (Fu et al., 2021).

Cardiac exosomes generated from hiPSCs improved swine MI recovery. These exosomes exhibited an upregulation in the expression of vascular endothelial growth factor and activation of the PKA signaling pathway, hence leading to a heightened angiogenic response in HUVEC (Almeria et al., 2019). By enhancing myocardial viability and angiogenesis, ESC exosomes enhance cardiac function in mice with AMI (Wang et al., 2018). The use of exosomes derived from the neural stem cell line CTX0E03 has been found to decrease the size of infarcts in mice through their influence on the opening of permeability transition pores in mitochondria. Exosomes released by neural stem cells in a mouse model of MI regulate these pores, protecting cardiomyocytes against the disease (Crewe et al., 2021). The majority of exosomes produced from stem cells can transmit miRNAs through targeted cell-free treatment. Wang et al. demonstrated that exosomes derived from ADMSCs and containing miRNA-671 exhibited a reduction in MI through the inactivation of the TGFBR2/Smad2 axis (Wang et al., 2021b). According to Lai et al., miRNA-221/222 mediated the protective effect of ADMSC exosomes on ischemic cardiomyocytes (Lai et al., 2020). Hypoxic MSC-derived exosomes released miR-125b and decreased cell death in a mouse model (Joo et al., 2020). MiRNAs can also be delivered via exosomes for the treatment of myocardial ischemia. Exosomes protect ischemic myocardial muscle due to their highly effective targeting capability. Several cardioprotective factors, such as miR-210, miR-183-5p, miR-125b, miR-182, miR-150, etc., are transported via exosomes produced by MSCs.

### 6.2 Heart failure

MSC therapy has been shown to enhance the condition of patients with ischemic heart failure through multiple mechanisms. MSC therapy exhibits cardioprotective and antifibrotic properties. A polypeptide called CTRP9 enhances the retention and survival rates of cortical bone-generated stem cells by upregulating the production of superoxide dismutase 2 and 3. CTRP9-281 induces the generation of exosomes enriched with vascular endothelial growth factor A by activating cortical bone-derived stem cells. This process subsequently facilitates angiogenesis, cardioprotection, and antifibrotic effects (Liu et al., 2021b). By dysregulating let-7i-5p and miR-27b, cardiac stromal cell exosomes utilized in the treatment of heart failure impede cardiac protection. The potential therapeutic application of exosomes from trophoblast stem cells in mitigating doxorubicin-induced myocardial damage through the pathway involving let-7i/Yes-associated protein 1 suggests a promising avenue for the treatment of heart failure (Ni et al., 2020).

The detection of asymptomatic individuals with latent heart disease is crucial for early management and prevention, thereby reducing the risk of further development of atrial fibrillation and heart failure. The levels of exosomal miR-181c and miR-495 are elevated in a canine model of heart failure associated with myxomatous mitral valve disease (Zhou et al., 2020b). By enhancing cardiac remodeling and delaying the course of valvular disease, exosomal miRNA therapy has identified prospective therapeutic targets, such as an upregulation of miR-9 and a downregulation of miR-599 (Janjusevic et al., 2021). According to these investigations, the response and expression of exosomal ncRNAs change between the early and late stages of valvular heart disease. This indicates that an accurate diagnosis impacts various stages of cardiac damage.

### 6.3 Cardiac fibrosis

The regenerative capability of the adult mammalian heart is limited, rendering it inadequate for repairing or regenerating itself after an MI. Consequently, this condition ultimately results in the development of significant cardiac fibrosis. Although current treatment approaches, including pharmacological, mechanical, and physical therapies, have demonstrated enhanced survival rates and decreased recurrence of ischemic instances in patients with AMI, they do not possess the ability to regenerate damaged cardiomyocytes or eliminate the formation of fibrotic scars (Nunez-Gil et al., 2019). Because of the reparative reaction mediated by fibroblasts and myofibroblasts to ischemic cell loss, the post-MI is characterized by widespread cardiac fibrosis (Talman and Ruskoaho, 2016). The first fibrotic response of the heart is essential in reducing the risk of ventricular wall rupture and hence plays an essential role in preventing eventual heart failure. Numerous bioactive compounds have been discovered within EVs or exosomes, which play a crucial role in facilitating the survival and proliferation of preexisting cardiomyocytes, hence contributing to cardioprotection (Xiao et al., 2018), facilitating the process of angiogenesis (Zhu et al., 2022a), mitigating hypertrophy of the heart (Lu et al., 2023), Immunomodulation, or the reduction of inflammation (Zhu et al., 2022b), reducing the risk of cardiac fibrosis (Fu et al., 2023), restoring the bioenergetics of the myocardium and inhibiting ventricular arrhythmias (Dawkins et al., 2022).

The administration of exosomes produced from MSCs has been demonstrated to improve disease characteristics in many models of disease. In a rat model of MI, for instance, Teng et al. (2015) found that exosomes produced from MSCs considerably enhanced endothelial cell tube development, decreased infarct size, hindered T cell activities, and protected cardiac systolic and diastolic performance. To further improve exosome cardioprotective action, miRNAs can be overexpressed in MSCs. For instance, by inhibiting CaMKII, exosomes isolated from miR-214 overexpressing MSCs protected cardiac stem cells against oxidative stress (Liu t al., 2018b). Exosomes generated by MSCs overexpressing miR-133 reduced inflammation and cardiac fibrosis in a rat model of AMI (Chen Y. et al., 2017b). *In vitro* experiments showed that exosomes produced from MSCs overexpressing miR-126 exhibited a reduction in the production of inflammation factors induced by hypoxia. Additionally, these exosomes facilitated myocardial cell migration and microvascular generation. *In vivo* studies further revealed that these exosomes contributed to a decrease in cardiac fibrosis and the levels of inflammatory cytokines (Luo et al., 2018b). Exosomes produced from activated cardiac fibroblasts have the potential to mediate endothelial cell dysfunction and enhance endothelial function (Ranjan et al., 2021). The endothelial cells are markedly affected by fibrosis-associated miRNAs in the cargo of fibroblast exosomes containing transforming growth factor-β1, particularly when miR-200a-3p levels are elevated. The impact of exosomes derived from human amniotic fluid MSCs has also been investigated in the context of cardiac fibrosis. It represents an innovative prospective therapeutic approach for cardiac fibrosis as it promotes angiogenesis and exerts pro-angiogenic impacts on endothelial cells associated with the condition (Hu et al., 2021).

### 6.4 Cardiomyopathy

Exosomes are also employed in the therapeutic management of dilated cardiomyopathy and cardiomyopathy linked to Duchenne muscular dystrophy. Using CDC-derived exosomes, an investigation revealed that in swine models of dilated cardiomyopathy, miR-146a-5p reduced the production of inflammatory cytokines (Hirai et al., 2020). Because they contain the dystrophin gene mRNA, exosomes derived from allograft myogenic progenitors can transiently restore relative gene expression and enhance cardiac function in mice (Su et al., 2018). The impact of allogenic exosomes on cardiomyopathy is beneficial. According to Wang et al., in the context of type 2 diabetes, cardiomyocytes demonstrate an anti-angiogenic role in rats with this condition. This outcome is attained via the translocation of miR-320 into endothelial cells via exosomes (Wang et al., 2014). Exosomes generated from non-stem cells can facilitate intercellular communication and confer cardioprotective benefits. Exosomes produced by cardiomyocytes, for instance, were discovered to stimulate endothelial cell angiogenesis in a glucose-deprivation setting (Garcia et al., 2016). Exosomes derived from myogenic progenitor cells alleviate diabetic cardiomyopathy and fibrosis-induced cardiac damage by inhibiting the TGF-β1/Smad2 signaling pathway. Diabetes mellitus is complicated by the transfer of exosomes containing Mst1 from microvascular endothelial cells of the heart to cardiomyocytes (Lin et al., 2019). Experimental evidence suggests that reducing inflammation and fibrosis by targeting macrophages generated from mouse bone marrow containing human antigen R associated with exosomes may be useful in the treatment of diabetic heart disease (Govindappa et al., 2020).

## 7 Conclusion and future prospects

In conclusion, exosomes emerged as important mediators of intercellular communication, demonstrating a wide array of potential uses in the physiological and pathological mechanisms of CVD. However, for exosomes to reach their maximum potential, sensitive detection, accurate characterization, and efficient isolation are required. Exosomes are crucial in the treatment and diagnosis of CVD. In addition, exosomes have the potential to serve as biological vehicles for the delivery of therapeutic genes and drugs in the treatment of CVD. Stem cell-derived exosomes can specifically stimulate angiogenesis, inhibit apoptosis, and alleviate stress-induced damage. Furthermore, bioengineered exosomes can improve their targeting capability, resulting in a rise in accumulation inside the cardiovascular system. This demonstrates significant promise as a customized therapeutic strategy for enhancing outcomes in patients with CVD.

Exosomes exhibit significant potential as vehicles for drug delivery due to their inherent membrane stability and targeted characteristics. Exosomal ncRNAs (circRNA, lncRNA, and tRF) are difficult to detect due to the presence of confounding interfering factors; hence, their therapeutic utility is limited. Exosomes are regarded as a natural drug delivery method, even though they can be modified to contain ncRNA for gene transmission or be modified with homing peptides to enhance cardiac targeting. In comparison to stem cell therapy, which carries the danger of immunogenicity and tumorigenicity, modified exosomes provide the potential for therapeutic utilization in CVD treatment. However, significant progress is still required to improve the loading efficiency and targeted modification of exosomes. This includes enhancing targeting and stability as well as refining cell pretreatment techniques (Wang et al., 2021c). Notably, there are still obstacles to overcome regarding the use of exosomes for gene or drug delivery. Several challenges persist in the field, including a poor extraction rate, the difficulty in achieving high concentrations and high purity of exosomes, as well as concerns regarding their long-lasting efficacy and safety. The potential impact of targeted modification on the structural integrity of exosomes during large-scale synthesis remains uncertain. Since *in vitro* and *in vivo* findings support the methods, ongoing research is conducted to resolve the difficulties. Current research focuses on optimizing administration routes, preparing formulations with excellent *in vivo* stability, improving target specificity, engineering exosomes without altering their biophysical and biochemical properties, etc. As a result, scalable manufacturing procedures are necessary to produce exosomes quickly, efficiently, and reliably if they are to be used as therapeutic carriers. Mass production of exosomes can be achieved by many techniques, including polymer-based precipitation, aqueous twophase systems, size exclusion chromatography, and ultrafiltration (Charoenviriyakul et al., 2017). The reaching large-scale desired exosomes for clinical usage is a main challenge.

Emerging technologies are crucial in enabling the detection and tracking of exosomes, as well as *in vivo* monitoring and analysis (Charoenviriyakul et al., 2017). Optical sensors and magnetically labeled particles hold tremendous potential for exosome detection and *in vivo* monitoring (Liu T. et al., 2020b). Since exosome technology is already being utilized more in the diagnosis and treatment of disease, researchers are encouraged to focus more of their efforts on overcoming the obstacles and limits in the field (Verweij et al., 2019). Several challenges hinder the progress of implementing exosomes in clinical applications. These include the absence of standardized protocols for isolating and analyzing these microvesicles, as well as the variability in yield caused by factors such as extraction methods, human error, storage conditions, and equipment type (Yang et al., 2023).

Native exosomes possess a vast array of proteins and nucleic acid constituents, which exhibit inherent therapeutic properties, hence classifying the exosomes as biological products. Despite the clinical validation of exosomes, regulatory agencies have yet to grant approval to any exosomal products (Whitford and Guterstam, 2019). Another crucial obstacle in exosome drug delivery is to guarantee that exosome-based therapies fulfill the criteria set by regulatory authorities to ensure clinical approval. Compared to other types of nanomedicines, the regulatory considerations for exosome-based therapeutics have not been tackled thoroughly to date. Although research and clinical studies related to the use of exosome-based therapeutics for drug delivery are still in their infancy, the on-demand methods used for advanced understanding and systemic characterization of exosomes will address the challenges and clinical transition issues of exosome-based therapeutics. Scalable cell culture conditions and methods for isolating and purifying exosomes are necessary for the effort to put therapeutic exosomes into industrial-scale production and clinical application. Due to high development expenses and the uncertainty of meeting regulatory and marketing standards, the use of large-scale stem cell cultures could potentially impede the production of stable and effective products. Similarly, there are few opportunities for supplying a large amount of cell-culture-conditioned medium to scale up exosome synthesis. To determine the optimal scaling-up conditions for therapeutically pertinent exosomes, it has proven challenging to obtain a satisfactory yield of high-purity exosomes during large-scale production. It is imperative to direct attention to the effectiveness and safety of exosomes to facilitate their utilization in clinical settings. The number of registered experiments conducted on exosomes and EVs is fewer than 200 (Stine et al., 2020). Exosomes derived from MSCs are scalable to extremely high and clinically relevant concentrations, according to preclinical data. The therapeutic benefits of exosomes derived from MSCs have been validated in numerous preclinical investigations across a wide range of surgical subspecialties (Butreddy et al., 2021). Exosomes have been found and employed as diagnostic biomarkers in numerous clinical trials. Beyond their use as stem cell produced exosomes, in numerous clinical trials, exosomes have been shown to serve as both therapeutic agents and biological markers. A comprehensive search was conducted on ClinicalTrials.gov, utilizing the keywords ‘exosome therapy’, ‘exosome treatment’, and ‘exosome’ to yield 188 records. However, of these, only 60 (or 32%) relate specifically to interventional studies that use exosomes as therapeutic agents (Tan et al., 2024). In December 2019, a clinical trial using umbilical stem cell (UMSC) exosomes to treat dry eye symptoms was registered (NCT04213248). At present, a clinical trial (NCT02138331) is being conducted to assess the impact of repetitive intravenous infusions of MSC exosomes derived from UCB blood on individuals with T1DM (Lee et al., 2021). Multiple clinical trials have been conducted on patients to validate the clinical advantages. Of these seven published clinical studies and fourteen ongoing clinical trials (as of September 2022) have evaluated MSC-derived exosomes as a therapeutic agent against various diseases, including stroke, Alzheimer’s disease, type 1 diabetes, kidney diseases, acute respiratory distress syndrome (ARDS), graft-versus-host disease (GvHD), and osteoarthritis (Lotfy et al., 2023). Exosome-based clinical trials are currently in progress. Exosomes generated from dendritic cells and mesenchymal cells are the primary types of exosomes utilized in clinical trials. However, the lack of ideal procedures for exosome manufacturing, isolation, and storage limits the clinical application of mesenchymal-cell-derived exosomes. To assess the effectiveness of exosome therapy, reliable characterization techniques, administration schedules, and cell culture conditions must be developed (Mendt et al., 2019). The identification of urinary exosomal proteins as a means of diagnosing autoimmune thyroid heart disease, as well as the use of exosomes derived from epicardial fat as atrial fibrillation biomarkers, have been explored. Clinical trials have been conducted, and ongoing recruitment efforts are focused on exploring the potential of exosomal miRNA in patients with MI. Despite the aforementioned limitations, exosomes have demonstrated a wide array of potential regarding the ongoing advancement of cell-based drug delivery systems and the clinical use of nano-biomimetic drug delivery systems. The potential success of clinical trials related to the treatment of exosomes has yet to be determined.
